# Facial lifting with Aptos Methods

**DOI:** 10.4103/0974-2077.41149

**Published:** 2008-01

**Authors:** M Sulamanidze, G Sulamanidze

**Affiliations:** *Clinic of Plastic and Aesthetic Surgery, Total Charm, Tbilisi, Georgia*

**Keywords:** Aptos, facelift, threadlift

## Abstract

Face lift hitherto had been a complicated and elaborate procedure. The introduction of threads to lift skin has been a major advance in lifting sagging skin. These threads which have barbs on their surface have simplified the procedure and have the possibilities of combination with other rejuvenation procedures. The article traces the evolution of threadlift.

## INTRODUCTION

Accumulated experience of past years with aggressive and serious invasive surgeries for face lifts, with their resultant complications have lead to research about finding answer to one question: Is it possible to lift soft ptotic tissues in lesser volume as well as less radically, with rather smaller scale invasion? We have been seriously dealing with this problem since 1988 and describe here the evolution of this minimally invasive face lift method.

*Early years*: Here are the main objectives that we first set to ourselves:

- to conduct research for the possibility of shifting various layers of soft facial tissues between each other or entirely, without cuts and surgical mobilization
- to examine possibilities of reducing skin space, its retraction and adaptation without excision
- to develop a new technique of low invasion surgery and manipulation as well as materials and tools for conducting such interventions
- to study the results of such invasion and create new refined methods

The very first thing that we decided to do was - to uplift the eyebrow tail. For that we have used thick silk, which we have pulled subcutaneously with the regular long needle and in the area of the temporal fascia. But methodology turned out to be unsound due to necessity of making a incision to visualize the temporal fascia, impression on the skin of the needle entry and exit points, presence of only one vectorial direction of tension leading to fast weakening of the bundle, sliding of tissues along threads and as a consequence, short-term results (1-3 months).

However, this preliminary experience provided evidence that layers of soft tissues shifted easily and skin, despite its rugosity, gradually fell out during first days after the surgery.

Another important observation was that short-term and weak effect of lifting was due to the fact that soft tissues were involved in only three points of stitches.

These observations encouraged us towards the idea of creating threads with prominences, which would be able to provide equal, multi-point linkage with subcutaneous tissues on its entire length.

**Figure 1:**
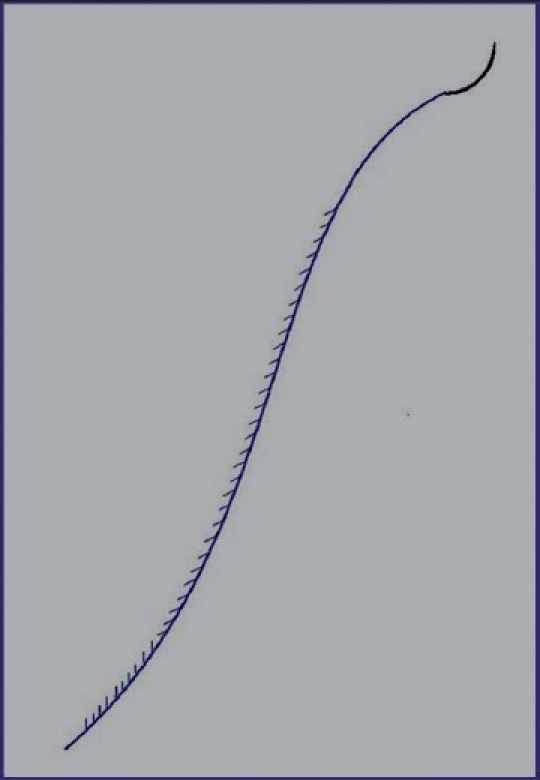
Aptos Suture, scheme of the wound suturing

Threads with prominences have been used for a very long-period of time - for suturing sinew (ligament, tendon, muscle) and wound edges; however, doctors of our clinic were the first to introduce threads with prominences for continuous suturing of surgical wounds and proposed them for lifting of flabby, ptotic soft tissues, as well as for rejuvenation surgeries. We called such threads Aptos (anti-ptosis)^1^. The first threads with incisions (Aptos Suture) for suturing operational and traumatic wounds with continuous stitches were introduced in 1998 [Figure 1].

Later the same name was adopted to all products and technologies of non-invasive lifting, which were developed and introduced by us into practice.

**Figure 2:**
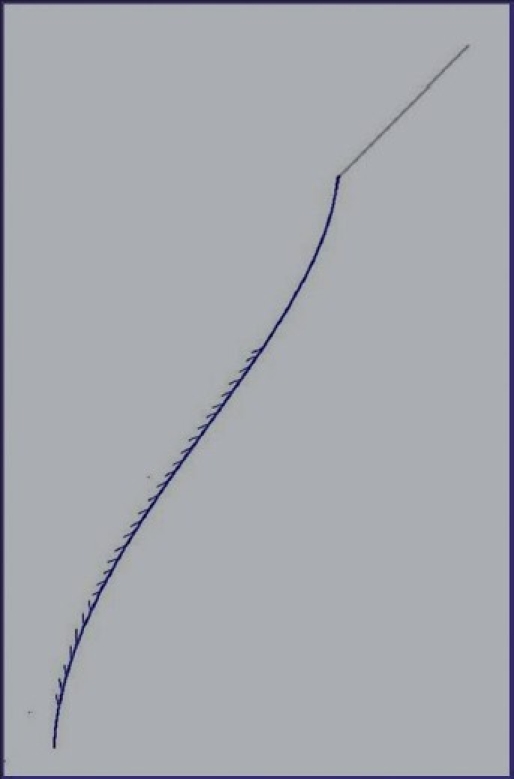
Threads with unilateral prominences, experiment of 1998

The early threads were threads with unilateral prominences, which were attached to a long needle. According to markings made on the skin, we would pull several threads subcutaneously through the small cut in the temporal region, where the lower remainder of the thread together with the needle would be cut off after pulling it out off the skin and the upper end after moderate lifting would be sutured to the fascia of the temporal muscle [Figure 2]. The same method was used to lift soft tissues of the sub-maxillary and cervical areas with fixing the upper end of the thread to the periosteum of the mastoid. These methods were not fully satisfactory, the results were not long-lasting and also required cuts.

**Figure 3:**
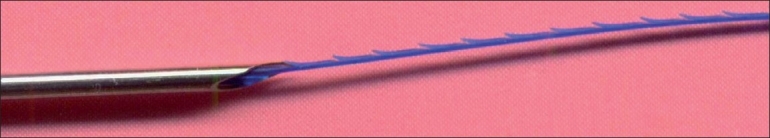
Aptos Thread enlarged - needle - conductor with the thread

Later, a needleless thread was created; it had converging prominences and could be introduced subcutaneously through a conducting needle; it also needed a more simplified manipulation, without needing a significant incision [Figure 3]. Accordingly, the optimal skin marking was developed for each area of the face, with full consideration for different anatomical, functional and pathological features of the different areas and pathologies. This technique of thread lifting became popular very soon and came to be called the Aptos Thread.

**Figure 4:**
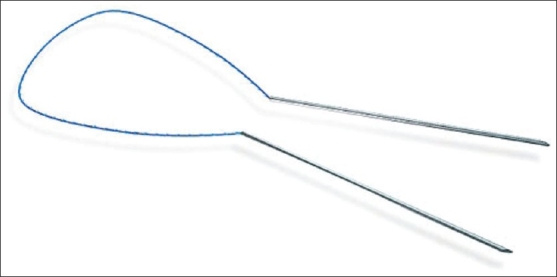
Aptos Thread 2G

However, in recent years (in 2002) we have returned to the idea of using a single product - a needle with thread, but the suggested suturing material was different from previous modifications by two needles to which one thread end was attached; the thread had diverse prominences and needles were paired by temporary commissure; thus their spikes would constitute a single point. Such device allowed for double increase of the thread lever and, therefore, allowed for increase of power and lifting stability. The idea of paired needles and new surgery techniques was an important innovation, which allowed for positioning threads without cuts, via simple puncture, since surgeons received an opportunity to introduce two needles with a single point, split them subcutaneously at the needed depth and pull them in different directions afterwards. This method of surgical invasion and the relevant product was called the Aptos Thread [Figure 4].

**Figure 5:**
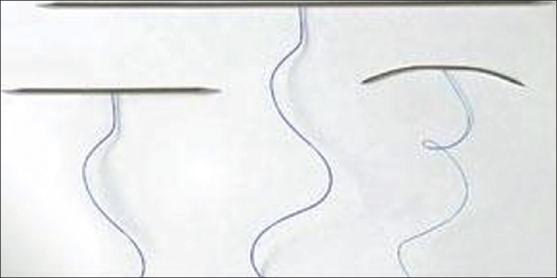
Aptos Needle, various modifications

It was later noticed that in some cases of rejuvenation and lifting surgeries, the threads with prominences alone, did not always lead to desirable long-lasting outcomes and additional lift and suture by smooth threads was also needed. Therefore, in the next stage of our research, a double-edged needle with the smooth suturing thread, attached to the needle in its central area - called Aptos Needle was created in 2003. This thread allows for double patency and also ensures introduction of the thread underneath the skin and subcutaneous suturing of soft tissues, without a skin retraction or a lifted stitch [Figure 5]. Further Aptos Needle modifications allowed for development of effective non-invasive aesthetic surgeries for suturing and lifting of soft tissues of the cheek, submaxillary and cervical areas (2003), chin, ptotic breast (2004) and slack shoulder tissues (2005).

**Figure 6:**
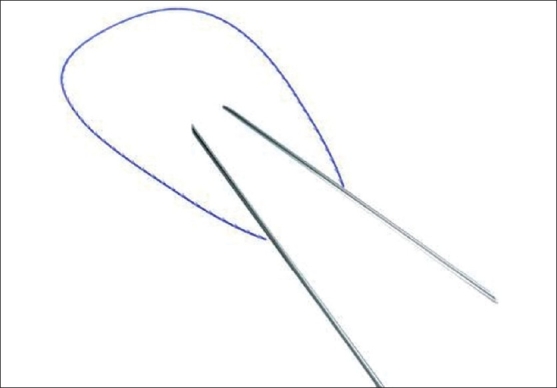
Aptos Needle 2G

The latest innovation in this series is the Aptos Needle 2G, which is more stable and radical than the previous Aptos Needle^2^. Aptos Needle 2G, has one thread with diverse prominences and two needles; these needles are double-edged and the thread is connected to them in the central area, as in case of the Aptos Needle [Figure 6].

**Figure 7:**
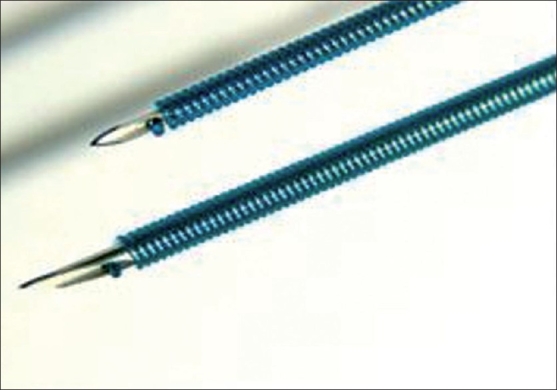
Aptos Springs

Lifting of facial areas with active muscular movements such as perioral area presented special challenges. For such cases, (lifting of so-called sadness fold or jowls), the principle of elastic lifting was developed in 2003. Elastic lifting is achieved by spring threads called Aptos Springs, made of polypropylene, which keep "memory".^3^ Aptos Springs are able to lift and fix soft tissues and at the same time also stretch and contract together with muscles. The lifting effect of such springs is supplemented by fibrous tissue accrued around threads [Figure 7].

## FACTORS WHICH DETERMINE THE OUTCOME OF SUCCESS IN THREADLIFT

These various developments and the resultant experience have lead to elucidation of certain principles which determine the outcome of success in thread lifting.^4^,^5^ These factors are:

1. Correct understanding of the Aptos lifting concept
2. Knowledge of topographic anatomy, especially anatomy of face
3. Consideration of the facial muscles kinetics
4. Knowledge of the pathogenesis of ageing and ptosis of soft tissues and
5. Correct determination of indications.

These factors are now further described in detail.

### Correct understanding of the aptos thread concept

Prominences on threads are located in every 0.5 mm and each of them is responsible for its own tissue area. Number of prominences on each thread is about 20. This is why Aptos lifting is even and uniform along the line. This is the major difference of Aptos from classical and endoscopic lifting methods, where mobilized tissues (skin, Subcutaneous Muscle Aponeurosis (SMAS)) are lifted and hung only to points fixed by stitches.

### Knowledge of topographic anatomy

It is known that facial and cervical layers of soft tissues can be shifted relative to each other, but only in specific and different blocks and areas. For instance, it is easy to shift skin of lateral areas of the forehead, eyebrow tail, mental area, cheek and malar areas. At the same time, any attempt to shift tissues of the posterior area of the face above the malar bone and its arch perimeter is difficult since in this area the skin is closely tied to the periosteum.

### Consideration of the facial muscles kinetics

It is not advisable to lift soft tissues in the active muscle area, especially vectorially; it would counteract the muscle work. Such areas need elastic lifting by Aptos Springs as outlined above.

### Knowledge of the pathogenesis of facial ageing

It is well-known that soft tissues ptosis is of a focal nature; some areas sag more than others. Some areas are resistant to sagging as they are held up by bundles (raper points) of connective tissues, vessels and nerves. Therefore, it is logical to lift in places, where these anatomic formations do not create any impediments.

### Correct determination of indications

The same methods shall not be advised to all and everybody. It is important to assess the suitability of different aesthetic techniques and then recommend the most suitable method or combination of methods applicable in a specific patient.

## CHOOSING THE APPROPRIATE METHOD IN DIFFERENT AREAS^1^

The different methods for face lifting for different indications are as follows:

### For eyebrows

- Aptos Thread method for mild light lifting
- Aptos Thread 2G method for more stable lifting

### For cheekbone areas

- Aptos Thread method for mild lifting, elimination of plaintive furrow and creation of high volume
- Aptos thread 2G method for more stable lifting
- Aptos Needle method for additional stable lifting, elimination of plaintive furrow and creation of high volume
- Aptos Needle 2G method for additional stable lifting, elimination of plaintive furrow and creation of high volume

### For marionette lines

- Aptos Spring method

### For mental areas

- Aptos Thread method (light lifting),
- Aptos Thread 2G method (more stable lifting),
- Aptos Needle 2G method (additional stable lifting).

### For sub-maxillary areas

- Aptos Needle method,
- Aptos Needle 2G method

## SIDE EFFECTS^4^

**Figure 8:**
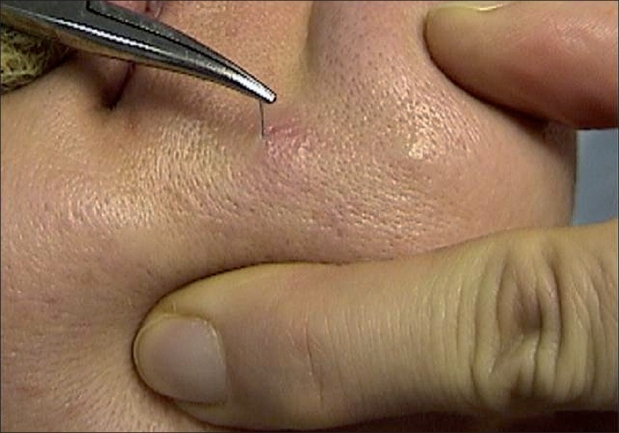
Exposure of the thread
- linear haemorrhage along the thread
- retraction of the skin in entry and exit points of needles and
- short-term (up to 3 months) and unstable results

While threadlift is a relatively safe method, side effects can occur, particularly if all points described are not given proper consideration. The possible side effects which are specific to threadlift include:

- breach of threads due to unilateral weakening of prominences
- abruption of threads to the skin surface, their migration
- asymmetry, hyper-correction, thread visualization [Figure 8]

**Figure 9:**
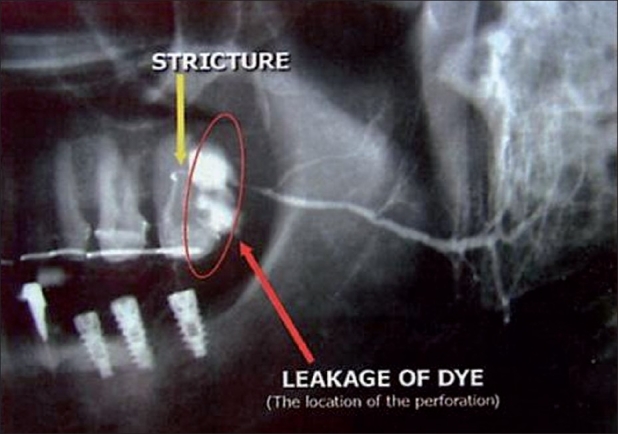
Breach of the *ductus parotideus*

In addition, other infrequent complications which may occur include inflammation of surrounding tissue, suppuration, damage to the parotid duct [Figure 9], branches of the facial nerve and regional nerve and vessels.

**Figure 10:**
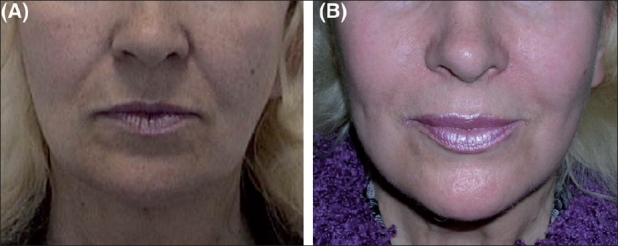
(A) Before aptos, (B) After aptos

**Figure 11:**
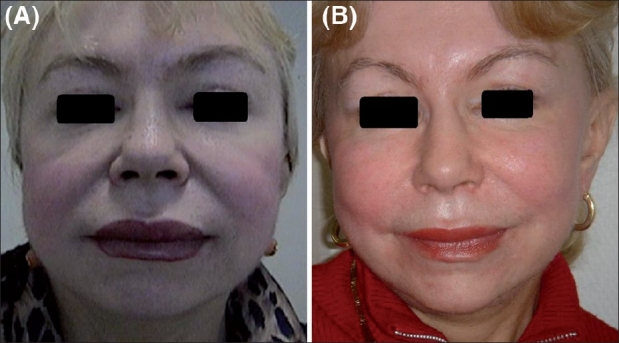
(A) Before aptos, (B) After aptos

Results of aptos are shown in Figures 10 and 11.

## CONCLUSIONS

Threadlift, as a anti-ageing procedure is gaining more popularity as it is a simple, minimally invasive procedure that can be done with local infiltrative anesthesia. Short healing time, reliable and long lasting effects and the possibility of combining with other aesthetic procedures such as Botox^®^, fillers, lasers, peels and liposuction are added advantages.

However, it should also be emphasized that threadlift is a relatively new procedure and like all new procedures, it requires further study and development, for proper evaluation.
